# Epidemiology of adolescent and young adult cancer and associated disparities in cancer pattern and care in India: findings from Varanasi’s population-based cancer registry, 2017–2019

**DOI:** 10.1007/s10552-025-02030-2

**Published:** 2025-07-12

**Authors:** Divya Khanna, Rajesh Vishwakarma, Anand N. Sharma, Atul Budukh, Rahul K. Verma, Aman Riguvanshi, Fahad Mahmood, Pankaj Chaturvedi, Satyajit Pradhan

**Affiliations:** 1 Department of Preventive Oncology, Mahamana Pandit Madan Mohan Malaviya Cancer Centre (MPMMCC) and Homi Bhabha Cancer Hospital (HBCH), Tata Memorial Centres, Varanasi, Uttar Pradesh 221005 India; 2 Homi Bhabha National Institute, Training School Complex, Anushakti Nagar, Mumbai, Maharashtra 400094 India; 3 Varanasi Cancer Registry, Mahamana Pandit Madan Mohan Malaviya Cancer Centre (MPMMCC) and Homi Bhabha Cancer Hospital (HBCH), Tata Memorial Centres, Varanasi, Uttar Pradesh 221005 India; 4 Centre for Cancer Epidemiology, Tata Memorial Centre, ACTREC, Navi Mumbai, Maharashtra 410210 India; 5 Department of Surgical Oncology, Tata Memorial Hospital, Mumbai, Maharashtra 400012 India; 6 Advanced Centre for Treatment, Research and Education in Cancer (ACTREC), Tata Memorial Centre, Mumbai, Maharashtra 410210 India; 7 Department of Radiation Oncology, Mahamana Pandit Madan Mohan Malaviya Cancer Centre (MPMMCC) and Homi Bhabha Cancer Hospital (HBCH), Tata Memorial Centres, Varanasi, Uttar Pradesh 221005 India

**Keywords:** Rural population, Sex, Health care disparities, Tobacco control, Early detection of cancer

## Abstract

**Introduction:**

Adolescents and Young Adults (AYAs) aged 15–39 represent a unique oncology demographic due to their distinct developmental needs. However, there is limited data on cancer burden and care disparities among the Indian AYAs. This study examines the burden, pattern, and disparities among AYAs in Varanasi, Uttar Pradesh.

**Methods:**

This study collected data from Varanasi's population-based cancer registry (PBCR) between 2017 and 2019. Data were analyzed for demographic, clinical, and cancer-related variables. Sex and site-specific crude, age-adjusted, and truncated rates for incidence and mortality per 100,000 population were calculated. Disparities in cancer pattern for age, sex, cancer site, and geographical region, sociodemographic and cancer care characteristics were assessed using standardized rate ratios and multivariable regression. Adjusted ratios with 95% confidence intervals were calculated. AAIR of the leading cancer site was compared with 24 Indian PBCRs from Cancer Incidence Five data and GLOBOCAN (2022) data.

**Results:**

Of 6821 cancer patients, 1105 (16.2%) were AYAs. The truncated age-adjusted incidence rate (AAIR) was 21 per 100,000 population, with mouth (16.5%), breast (12.2%), and tongue (6.2%) cancers leading. Oral cancer was most common cancer in male AYAs, with truncated AAIR of 9.2 per 100,000, ranking third highest among Indian PBCRs. Females had higher risks of stomach, gallbladder, and thyroid cancers. AYAs were more likely to access diagnostic and definitive treatment but faced income and employment vulnerabilities when compared with adults aged ≥ 40 years.

**Conclusion:**

This study highlights significant cancer burden and disparities among AYAs in Varanasi. Targeted screening, tobacco control policies, and region-specific interventions, especially social support schemes, are crucial to addressing these inequities and improving outcomes.

**Supplementary Information:**

The online version contains supplementary material available at 10.1007/s10552-025-02030-2.

## Introduction

The Adolescent and Young Adult Oncology Progress Review Group defines Adolescents and Young Adults (AYA) as individuals aged 15–39 [1]. This group represents a unique and challenging subset within oncology because of the significant psychosocial and physical development during this period [2]. Cancer diagnosis in AYAs disrupts key milestones like education, career, relationships, and family planning [3].

GLOBOCAN 2022 estimated 1,321,779 AYA cancer cases globally, with China, India, and the US contributing 23.9%, 11.3%, and 6.5%, respectively [4]. Cancer is the leading cause of death among the AYAs, excluding homicide, suicide, and unintentional injury [5]. The distribution of cancers varies significantly across different age subgroups within the AYA category [2]. Their biological and genomic characteristics often differ from those seen in older adults (aged ≥ 40 years), posing additional challenges in treatment and management [2].

Globally, AYAs with cancer face unique challenges, including infertility, body image concerns, and disrupted relationships and family planning. These issues, along with financial burdens—especially for older AYAs—impact quality of life (QoL). Psychosocial support and multidisciplinary care, including mental health and reproductive services, are essential to address their needs. Despite such efforts, AYA survivor’s significant disparities and remain at risk for late effects, recurrence, and second malignancies. Unlike children and older adults, AYA survival rates have stagnated over two decades due to low clinical trial participation, distinct disease biology, and barriers like delayed diagnosis and limited insurance coverage [1–3, 6].

In low- and middle-income countries (LMICs), such as India, the burden of AYA cancers is compounded by limited resources and healthcare infrastructure [7]. A recent Indian study analysed data on primary site cancers among AYAs from 28 Population-Based Cancer Registries (PBCRs) and 58 Hospital-Based Cancer Registries (HBCRs) under the National Cancer Registry Programme for the reporting year 2012–2016 [7]. The study reported a median age-adjusted incidence rate (AAR) of 22.2 per 100,000 population among males and 29.2 per 100,000 population among females. A significant increase in cancer incidence was observed among AYA males between 1985 and 2015, while a non-significant decline was noted for females. The projected number of cancer cases in both sexes is expected to increase to 178,617 by 2025. Most of these cancers were in the loco-regional stage at the time of diagnosis [7].

In the Indian context, sociodemographic factors such as age, sex, education, socioeconomic status, and geographic location play a pivotal role in determining cancer diagnosis, access to care, and health outcomes. Regional variability in healthcare infrastructure, levels of awareness, and social vulnerabilities can contribute to diagnostic delays and restricted access to treatment, particularly in resource-constrained settings. These disparities underscore the necessity of understanding how sociodemographic and contextual factors influence the burden of cancer and the provision of care, especially among AYA patients, for whom evidence from India remains scarce [7–11]. This study seeks to address this gap by examining these factors in the Varanasi district, generating findings that may inform targeted strategies to improve cancer diagnosis and care for AYA populations, both in the study setting and in comparable contexts across India. Despite the critical need for such research, there is a paucity of literature examining the epidemiology and disparities in cancer burden and care in the Indian population.

The Varanasi PBCR, established in 2017, indicates the cancer burden in Uttar Pradesh, the largest state in India. With this background, using data from the PBCR for the period 2017–2019, the present study aims to describe the burden of cancer among AYA patients in Varanasi district, and to examine disparities in cancer patterns by age, cancer site, sex, and geographical residence. The study also aims to assess disparities in sociodemographic characteristics and cancer care between AYA patients and adults aged ≥ 40 years.

## Methods

### Study design and settings

This study is a retrospective population-based cohort study that utilized the data from the Varanasi PBCR. This PBCR was established in 2017, as a part of PBCR network administered by Tata Memorial Centre (TMC), Mumbai. This registry provides crucial data on cancer burden in Uttar Pradesh. The TMC collaborates with district health administration to facilitate cancer registration in the defined area [8, 12].

The estimated population of Varanasi is approximately 4 million (4,005,176), with a predominance of rural inhabitants (57%) spread over an area of 1535 square kilometers. The district comprises eight rural blocks containing 1,295 villages and 90 urban wards, many of which are not sufficiently covered by conventional cancer screening programs. Varanasi operates under a three-tier healthcare delivery and referral system established by national health mission. The district houses two government institutions-supported tertiary cancer care centres (TCCs) and a few private centres, all predominantly located in urban areas [8, 12].

### Study participants and data source

The study participants included all registered cancer patients aged 15 years and above residing in Varanasi. Cancer incidence and mortality data from 2017 to 2019 were sourced from the Varanasi PBCR. Data on cancer patients were actively collected from hospitals, medical colleges, radiology and pathology diagnostic centres, birth and death registration office, primary and secondary health care centres, frontline healthcare workers, and the community [8, 12]. The information included socio-demographic and cancer-related variables from various sources, using a standardized proforma developed by [8, 12]. The PBCR registration proforma was developed based on the 35 data elements encompassing sociodemographic and cancer-specific information, as outlined by MacLennan et al. [13, 14]. The collected information was entered into CanReg5 software developed by the International Agency for Research on Cancer (IARC), after review of the existing PBCR database [8, 12]. Trained field investigators conduct these activities and are periodically monitored by faculty from the Centre for Cancer Epidemiology, TMC, Mumbai. Quality control measures include systematic and random checks, removal of duplicate entries, re-abstraction of 5% of randomly selected cases, staff retraining, and calculation of data quality indices for completeness [8, 12].

### Measures

The collected data encompassed demographic details, including age, sex, geographical residence (rural/urban as per the Census, India classification [8]), religion (Hindu/Muslim/Christian/Sikh/Buddhist/others), self-reported education levels (illiterate/literate/up to primary/up to secondary/ up to technical after 10th/graduate/post-graduate), mother tongue (Hindi/other languages), occupation (Professional, semi-professional, clerical, government and private employees/ Farmer, skilled, semi-skilled, unskilled workers/ Unemployed, student, house-wife), and monthly income (lower/lower middle/ upper and upper middle). Tumour characteristics included topography and morphology (five-digit codes, where the first four digits indicate the specific histological term, and the fifth digit is the behavior code [malignant, benign, in situ, or uncertain]) of primary cancer site, basis of diagnosis (death certificate/clinical/radiology/specific tumour markers/cytology/histology of primary site/histology of metastasis/others), type of treatment received (surgery/radiotherapy/chemotherapy/combination of two or more definitive modalities/palliation/no treatment/others/unknown), treatment status (complete/ongoing/not completed/ongoing/unknown), and patient outcomes (alive/died/migrated/unknown), including mortality. Malignancies were classified per the International Classification of Diseases for Oncology, third edition (ICD-O) [8, 12–14].

### Statistical analysis

We computed sex—and site-specific cancer burden using crude, truncated, and age-adjusted rates (AAR) for incidence (AAIR) and mortality (AAMR) per 100,000 population. Additionally, we calculated cumulative risk, reflecting the probability of an AYA being diagnosed with cancer within the 15–39 years age group. The AARs were determined using the direct standardization method, with World Standard Population 2000 as the reference. The AAIR of the leading cancer site was compared with the AAIRs of other Indian PBCRs and with those of countries with the highest AAIRs for the same cancer site. For this, we utilized data from Cancer Incidence in Five Continents (CI5-Volume XII) [15] and GLOBOCAN (2022) [4] data, respectively.

Sex-specific disparities in cancer incidence and mortality were evaluated using Standardized Rate Ratio (SRR), defined as AAR in the female AYA population divided by AAR in the male AYA population, with 95% confidence intervals (CI). Urban–rural (geographical) disparities were assessed by calculating sex-specific AAIR according to their residence.

In this study, participants were categorized into two age groups: adolescents and young adults (AYAs, aged 15–39 years) and individuals aged 40 years and above. This classification was selected to underscore the distinct challenges faced by AYAs, who are navigating major life transitions related to education, employment, financial stability, and family planning. In contrast, adults aged 40 years and above are generally considered to have achieved greater stability in these domains. Although a more granular age group stratification (e.g., 40–49, 50–59 years) could potentially yield additional insights, the sample size limitations of this study precluded meaningful subgroup analysis. Thus, the dichotomous age grouping was maintained to align with the primary objective of examining disparities in cancer burden and care specific to AYAs within the study context.

To assess sociodemographic and cancer care disparities between AYAs and adults aged ≥ 40 years, univariable and multivariable regression analyses were conducted. Variables with a *p*-value of < 0.2 in univariable analysis were included in the multivariable model after assessment for collinearity [8]. Crude and adjusted odds ratios (ORs) with 95% confidence intervals (CIs) were calculated, with statistical significance set at *p* < 0.05. Data were entered and analyzed using Microsoft Excel and the Statistical Package for the Social Sciences (SPSS, version 21).

### Ethical considerations

This study utilized de-identified data from Varanasi’s PBCR, which is integrated into hospital services and routine public health surveillance to monitor the impact of cancer control programs within the district. According to national [16] and international [17] guidelines, such activities are exempt from ethical review by the review board. Consequently, this study did not require ethics approval or consent to participate. The research was conducted following the provisions of the Declaration of Helsinki regarding research involving human subjects. Neither patients nor the public were involved in the design, conduct, reporting, or dissemination plans of this research.

## Results

### Epidemiological profile of AYAs cancer patients in Varanasi

Of the 6821 patients registered under Varanasi PBCR from 2017 to 2019, 16.2% of incident cancers (1105) were from the AYA age group (Table 1). Among these AYA patients, more than half were males (604, 54.7%), and the majority had secondary or higher levels of education (761, 68.7%). Most of them were from rural backgrounds (560, 50.7%), were Hindu by religion (923, 83.5%), and spoke Hindi as their mother tongue (1046, 94.6%). Almost half (542, 49.0%) were unemployed, students, or homemakers, and most belonged to lower income levels (501, 45.3%) (Supplementary Table 1).

**Table 1:** Distribution of registered cancer patients according to the sex and age groups, 2017–2019 (n = 6821)

Age group (in years)	Frequency (Percentage)		
	Male	Female	Both sexes
0–14	116 (3.0)	40 (1.3)	156 (2.3)
15–39	604 (15.7)	501 (16.8)	1105 (16.2)
40–59	1639 (42.6)	1470 (49.4)	3109 (45.6)
≥ 60	1487 (38.7)	964 (32.4)	2451 (35.9)
Total	3846 (56.4)	2975 (43.6)	6821 (100)

### Burden and disparities in pattern among AYAs cancer patients in Varanasi

The overall AAIR for cancer among AYA patients was 21 per 100,000 population, and specifically among AYA males and females, it was 22.2 and 19.5 per 100,000 population, respectively. The overall AAMR for cancer-related deaths among the AYA patients was 9.3 per 100,000 population, and it was 10.6 and 7.9 for male and female AYA patients, respectively (Supplementary Fig. 1).

**Fig. 1:**
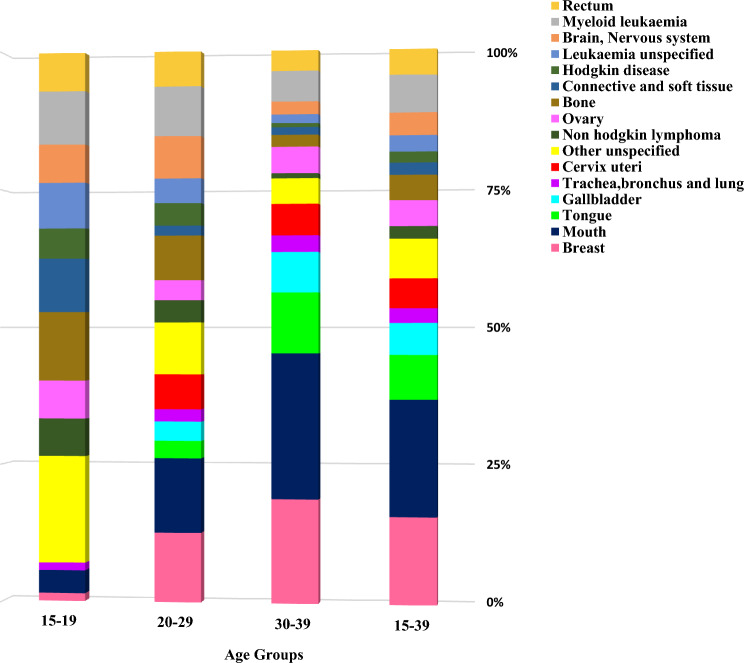
Proportions (%) of primary site-specific cancer incidence among the different age groups in Adolescent and Young Adult cancer patients in Varanasi, 2017–2019

Further analysis by age subgroups revealed that for males, the AAIR was 1.6 for ages 15–19, 6.6 for ages 20–29, and 14.0 for ages 30–39. When females were considered, the AAIR was 1.0 for ages 15–19, 5.5 for ages 20–29, and 13.0 for ages 30–39. (Supplementary Fig. 2). Whereas the incidence proportions for mouth, tongue, breast, uterine cervix, lung, and gall bladder cancers increased with age, the incidence proportions for ovarian, haematological, brain, rectal, bone, and soft tissue malignancies decreased (Fig. 1).

The overall leading cancers among AYA patients were from mouth (16.5%, AAIR 3.5 per 10,000 population), breast (12.2%, AAIR 2.6 per 100,000 population), tongue (6.2%, AAIR 1.3 per 100,000 population), myeloid leukaemia (5.2%, AAIR 1.1 per 100,000 population) and gallbladder (4.4%, AAIR 0.9 per 100,000 population) (Supplementary Table 2).

**Fig. 2:**
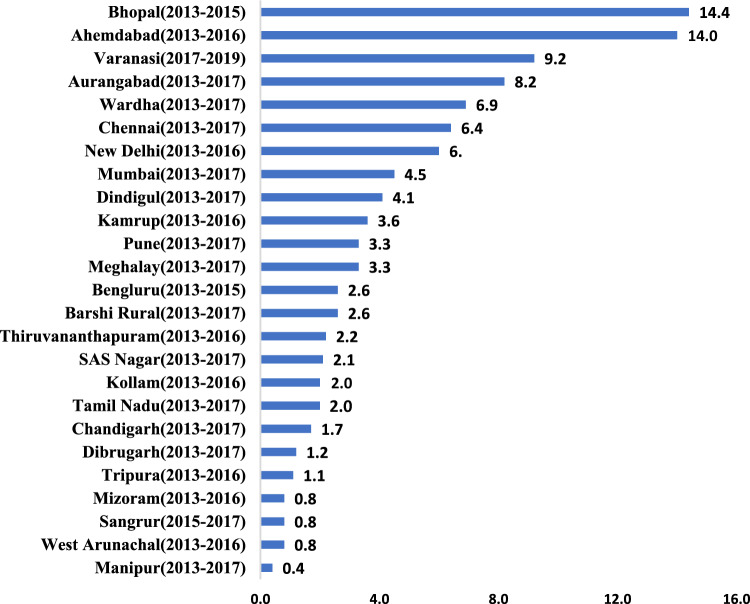
Age-adjusted Truncated Incident rates of oral cancer (C00-C06) among Indian Adolescent and Young Adult males in 25 population-based cancer registries

Among the AYA males, leading cancer sites were the mouth, tongue, myeloid leukaemia, rectum, and bone tumours with AAIR of 6.3, 2.3, 1.3, 1.0, and 1.0 per 10,000 population, respectively. (Supplementary Table 3) For AYA females, leading cancer sites were breast, cervix, ovary, gallbladder, and myeloid leukaemia, with AAIRs of 5.3, 1.8, 1.5, 1.4, and 0.9 per 100,000 population, respectively. (Supplementary Table 4) The lifetime risk of developing cancer (15–39 years) among AYAs was highest for the mouth and breast for males and females, respectively. (Supplementary Tables 5,6). Oral cancer (mouth and tongue; C00-C06) was the leading cancer site among male AYAs in Varanasi, with a truncated AAIR of 9.2 per 100,000 population. When compared to other Indian registries using CI5 data, Varanasi had the third- highest AAIR for oral cancer among 26 Indian PBCRs (Fig. 2) Additionally, AAIR for oral cancer in Varanasi male AYAs was significantly higher than the GLOBOCAN estimates for the top 15 countries with the highest AAIR for oral cancer in AYAs (Supplementary Fig. 3).

When compared to males, females have a higher risk of developing stomach cancer (2.67 times), gallbladder cancer (3.2 times), and thyroid cancer (3.8 times). Conversely, males have a significantly greater likelihood of developing cancers of the tongue (7.7 times), the mouth (10 times), and the pancreas (3.4 times). Moreover, males have approximately twice the risk of developing cancers of the colorectal, bone, brain, and central nervous system, as well as haematological malignancies, including Hodgkin and non-Hodgkin lymphoma and leukaemia (Table 2).

**Table 2:** Female-to-Male Incidence Rates and Standardized Rate Ratios (SRR) Incidence for the Leading Sites in Patients Aged 15 to 39 Years, 2017 to 2019

ICD code	Cancer Type	Male AAIR	Female AAIR	SRR	95% Confidence Interval	
All Cancer Combined		22.2	19.5	0.88	0.91	0.85
C01-C02	Tongue	2.3	0.3	0.13	0.16	0.11
C03-C06	Mouth	6.3	0.6	0.10	0.12	0.09
C07-C08	Salivary Glands	0.2	0.1	0.55	0.80	0.38
C11	Nasopharynx	0.0	0.1	2.41	5.04	1.15
C15	Oesophagus	0.1	0.1	1.52	2.41	0.96
C16	Stomach	0.2	0.5	2.67	3.52	2.03
C18	Colon	0.7	0.3	0.45	0.56	0.36
C19	Rectum	1.0	0.5	0.52	0.62	0.43
C21	Anus	0.1	0.2	1.36	1.93	0.95
C22	Liver	0.6	0.8	1.35	1.62	1.13
C23-C24	Gallbladder	0.5	1.4	3.19	3.76	2.70
C25	Pancreas	0.3	0.1	0.29	0.43	0.19
C30-C31	Nose, Sinuses	0.2	0.0	0.18	0.30	0.10
C32	Larynx	0.1	0.1	0.98	1.50	0.64
C33-C34	Trachea, bronchus and lung	0.5	0.3	0.59	0.74	0.47
C40-C41	Bone	1.0	0.4	0.39	0.47	0.32
C44,C47,C49	Connective and Soft Tissue	0.6	0.7	1.27	1.53	1.06
C64	Kidney	0.2	0.2	1.17	1.68	0.82
C67	Bladder	0.2	0.2	0.88	1.25	0.62
C70-C72	Brain, Nervous System	0.9	0.4	0.44	0.53	0.36
C73	Thyroid	0.2	0.7	3.82	5.00	2.92
C81	Hodgkin Disease	0.4	0.2	0.49	0.66	0.37
C82-C85,C96	Non-Hodgkin Lymphoma	0.5	0.2	0.39	0.52	0.29
C92-C94	Myeloid Leukaemia	1.3	0.9	0.74	0.86	0.64
C95	Leukaemia Unspecified	0.6	0.3	0.54	0.68	0.43
O&U	Other Unspecified	1.5	0.7	0.49	0.57	0.42

**Fig. 3:**
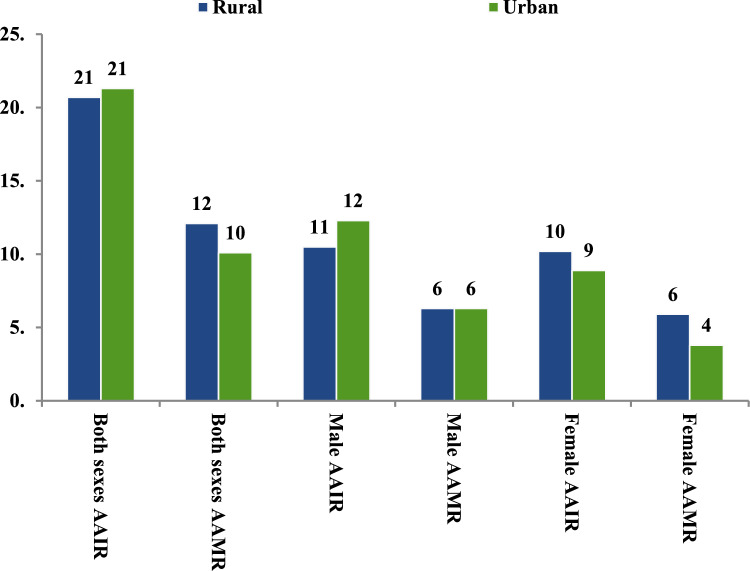
Age-adjusted cancer incidence (AAIR) and mortality (AAMR) rates (per 10,000) according to the geographical residence among Adolescent and Young Adult cancer patients in Varanasi, 2017–2019

Urban males had higher AAIR than rural males (12.3 vs. 10.5 per 100,000 population), but rural females had a higher AAIR than urban females (10.2 vs. 8.9 per 100,000 population). The AAMR was the same for urban and rural males (6.3 per 100,000 population) but higher in rural females compared to urban females (5.9 vs. 3.8 per 100,000 population) (Fig. 3).

### Disparities in sociodemographic and cancer care characteristics between AYA and adults aged ≥ 40 years

On multivariable regression analysis, AYA patients had 2 to 5 times greater odds of achieving higher education levels than older patients (aged ≥ 40 years). However, AYA patients exhibited lower odds of employment and achieving higher income levels compared to older patients. Additionally, AYA patients were significantly more likely to receive cancer confirmation through microscopic verification and were more likely to have completed or undergoing cancer treatment compared to older patients (Table 3).

**Table 3:** Multivariable regression analysis showing the differences in the distribution of socio-demographic and cancer-related variables between the AYA cancer patients and older patients aged ≥ 40 years (n = 6725)

Variables	40 years & above N (%)	15–39 years N (%)	Crude Odds ratio (95% CI)	P value	Adjusted Odds ratio (95% CI)	P value
**Sex**						
Female	2434 (43.8)	501 (45.3)	Ref			
Male	3126 (57.2)	604 (54.7)	0.94 (0.82–1.07)	.339	–	–
**Geographical residence**						
Rural	2756 (49.6)	560 (50.7)	Ref			
Urban	2804 (50.4)	545 (49.3)	0.99 (0.84–1.09)	.500	–	–
**Education**						
Illiterate	1267 (22.8)	110 (10.0)	Ref			
Literate	1258 (22.6)	217 (19.6)	1.99 (1.56–2.53)	.000	2.03 (1.58–2.60)	**.000**
Up to secondary	2157 (38.8)	507 (45.9)	2.71 (2.18–3.36)	.000	3.18 (2.54–4.00)	**.000**
Senior secondary	779 (14.0)	254 (23.0)	3.76 (2.95–4.78)	.000	5.24 (4.03–6.82)	**.000**
No information	99 (1.8)	17 (1.5)	1.98 (1.14–3.43)	.015	1.49 (0.74–2.98)	.264
**Mother tongue**						
Hindi	5154 (92.3)	1046 (94.7)	Ref			
Others	406 (8.7)	59 (6.3)	0.72 (0.54–0.95)	.020	0.81 (0.60–1.09)	0.164
**Religion**						
Hindu	4944 (88.9)	923 (83.5)	Ref			
Others	616 (11.1)	182 (16.5)	1.58(1.32–1.89)	< 0.01	1.88(1.55–2.27)	** < 0.01**
**Occupation**						
Unemployed, student, house-wife	2157 (38.8)	542 (49.1)	Ref			
Farmer, skilled, semi-skilled, unskilled workers	2334 (42.0)	393 (35.5)	.67 (0.58–0.77)	.000	.61(0.53–0.71)	**.000**
Professional, semi-professional, clerical, government, private employees	1004 (18.0)	156 (14.1)	.62 (0.51–0.75)	.000	.45 (0.36–0.56)	**.000**
No information	65 (1.2)	14 (1.3)	.86 (0.48–1.54)	.606	.79 (0.38–1.66)	.540
**Income**						
Lower	2420 (43.5)	501 (45.3)	Ref			
Lower middle	1926 (34.6)	353 (31.9)	.885 (0.76–1.03)	.109	.798 (0.68–0.93)	**.005**
Upper and upper middle	801 (14.4)	130 (11.8)	.784 (0.64–0.97)	.022	.669 (0.53–0.84)	**.001**
No information	413 (7.4)	121 (10.9)	1.415 (1.13–1.77)	.002	1.421 (1.11–1.82)	**.005**
**Basis of diagnosis**						
Clinical only	480 (8.6)	83 (7.5)	Ref			
Radiology	569 (10.2)	87 (7.9)	.88 (0.64–1.22)	.457	.99 (0.71–1.39)	.961
Verbal autopsy	967 (17.4)	133 (12.0)	.79 (0.59–1.07)	.129	.97 (0.71–1.32)	.832
DCO	31 (0.6)	5 (0.5)	.93 (0.35–2.47)	.888	1.08 (0.40–0.91)	.885
Histology	2959 (53.2)	654 (59.2)	1.28 (1.00–1.64)	.052	1.19 (0.92–1.53)	.192
Cytology	554 (10.0)	143 (12.9)	1.49 (1.11–2.00)	.008	1.42 (1.04–1.93)	.**026**
**Treatment type**						
No/ alternative treatment	518 (89.6)	60 (10.4)	Ref			
Any definitive treatment	1467 (83.0)	300 (17.0)	1.77 (1.31–2.37)	.000	1.29 (0.90–1.83)	.164
Multimodality treatment	2063 (81.5)	469 (18.5)	1.96 (1.48–2.61)	.000	1.27 (0.89–1.81)	.186
Palliative	1068 (85.1)	187 (14.9)	1.51 (1.11–2.06)	.009	1.42 (1.00–2.04)	.058
No information	444 (83.3)	89 (16.7)	1.73 (1.22–2.46)	.002	1.41 (0.90–2.21)	.129
**Treatment status**						
Not completed	2465 (44.3)	368 (33.3)	Ref			
Complete	1185 (21.3)	312 (28.2)	1.764 (1.49–2.08)	.000	1.518 (1.26–1.83)	**.000**
Ongoing	1109 (19.9)	284 (25.7)	1.715 (1.45–2.03)	.000	1.473 (1.23–1.76)	**.000**
Not applicable	233 (4.2)	28 (2.5)	.805 (0.54–1.21)	.296	1.053 (0.64–1.72)	.836
No information	568 (10.2)	113 (10.2)	1.333 (1.06–1.68)	0.014	1.204 (0.87–1.67)	.269

## Discussion

There is an increase in focus towards AYA oncology in India. Furthermore, a recent nationwide study on data from 28 PBCRs and 58 HBCRs from India reported significant increase in cancer incidence, especially among AYA males [7]. India, one of the largest countries with a highly diverse population, necessitates region-specific studies to understand the cancer burden and associated disparities. Such studies are crucial for presenting and formulating effective AYA-specific cancer prevention and control policies. To the best of our knowledge, this is the first study from India to analyze age, sex, geographical, and cancer care-related disparities among Indian AYA population. This study provides an in-depth analysis of the cancer burden among the AYA population in Varanasi district of Uttar Pradesh, India's largest state, utilizing data from PBCR for 2017–2019.

### Cancer burden among the AYAs

Out of 6,821 registered patients, 16.2% (1,105) were AYA, with a majority being male (54.7%) and half from rural backgrounds (50.7%). Compared to 28 PBCRs in India (2012–2016), the Varanasi PBCR (2017–2019) reported the sixth highest proportion of AYA cancers (16.2%), following regions such as West Arunachal (20%), Nagaland (19.7%), Pasighat (17.9%), Nagpur (17.4%), and Aurangabad (17.2%) [18]. North American data (2011–2015) reported 5% AYA cancers [2], while Japan (2016–2018) [19] and the European Union (2020) [20] reported 2% and 5%, respectively. India’s higher proportion of young population likely contributes to the higher AYA cancer burden [18]. Our findings highlight the need to prioritize AYA cancer surveillance in national cancer control programs, develop region-specific cancer registries and epidemiological studies to inform targeted strategies, and implement AYA-specific health education to raise risk awareness.

### Leading cancer sites among the AYAs

We observed that overall leading cancers among Varanasi AYA patients were mouth (16.5%, AAIR 3.5 per 10,000 population), breast (12.2%, AAIR 2.6 per 100,000 population), tongue (6.2%, AAIR 1.3 per 100,000 population), myeloid leukaemia (5.2%, 1.1 per 100,000) and gallbladder (4.4%, 0.9 per 100,000 population). However, as per GLOBOCAN (2022), leading cancer incidence sites among the Indian AYA for both sexes in terms of absolute numbers were breast (17.2%), lip and oral cavity (11.2%), cervix uteri (9.8%), leukaemia (8.4%) and thyroid (4.9%) [4]. Globally, the leading sites among AYA included breast (18.6%), thyroid (17.9%), cervix uteri (8.0%), leukaemia (4.9%) and colorectum (4.5%), non-Hodgkin lymphoma (3.9%) [4].

The observed pattern of cancer incidence among AYA patients in Varanasi highlights both local and broader epidemiological influences. Mouth cancer emerged as the leading cancer site, aligning with the high prevalence of smokeless tobacco (SLT) use, often combined with areca nut, deeply rooted in the local culture [8, 12]. Breast cancer, the second most common site, is consistent with national and global data, underscoring its significance as a leading malignancy among young women [4]. Haematological malignancies, such as myeloid leukaemia, also featured prominently, reflecting patterns reported in both Indian and global datasets specific for AYA populations [4]. Of particular note is the relatively high incidence of gallbladder cancer in this study. This finding may be attributed to multiple region-specific factors, including high mustard oil consumption, a known risk factor for gallbladder cancer, as well as the prevalence of cholelithiasis, chronic typhoid infection, and the consumption of contaminated snails, which may harbor liver flukes. Additionally, arsenic contamination of groundwater—a concern particularly in rural areas of the Varanasi region—has been increasingly recognized as a potential contributor to gallbladder carcinogenesis [8]. Previous studies from India, including population-based cancer registries and case–control studies, have also reported a higher incidence of gallbladder cancer in rural populations, lending support to these findings [8]. Overall, the cancer profile observed in Varanasi’s AYA population highlights the interplay of lifestyle, cultural, and environmental factors unique to the region, while also reflecting broader trends observed at the national and global levels.

While cancers like mouth, tongue, breast, and cervix can be managed through effective screening and early detection, India’s population-based screening coverage remains inadequate, particularly in Uttar Pradesh [12, 22]. This calls for research and practical strategies to improve screening reach and uptake. Although smoking rates have slightly declined, the continued high prevalence of smokeless tobacco and areca nut use remains a significant concern, especially in regions like Varanasi [23–26]. Additionally, cancers such as rectum, breast, ovary, and gallbladder have strong associations with obesity and metabolic diseases [27]. Alarmingly, a national survey found abdominal obesity rates of 32.2% (32.0–32.4) and 49.3% (49.0–49.5) in the 20–39 and 30–39 age groups, respectively [28]. Addressing these risk factors requires robust health education campaigns promoting lifestyle changes and strict enforcement of regulations like the Cigarettes and Other Tobacco Products Act (COTPA) [29]. Integrating cancer screening with existing adolescent health programs and establishing adolescent-friendly clinics could facilitate early detection and prevention of risk factors.

The AAIR increased with age, peaking in 35–39 age group, consistent with Indian and international studies. Incidence proportions rose with age for cancers of mouth, tongue, breast, uterine cervix, lung, and gall bladder, while it declined for ovarian, haematological, brain, rectal, bone, and soft tissue malignancies. Data from 28 Indian PBCRs showed higher rates of haematological cancers in the 15–24 age group and breast, mouth, and tongue cancers in the 30–39 age group [7]. Similar findings were reported in US [30] and Japanese [19] registry studies, with haematological, soft tissue, and central nervous tumours more common in 15–19-year-olds and breast, uterine cervix, and gastrointestinal cancers predominant in older groups.

### Disparities in cancer patterns among AYAs

Geographical disparities in cancer incidence and mortality among AYAs were notable. The overall AAIR was similar in urban and rural settings (21.3 vs 20.7 per 100,000), but mortality rates were higher in rural areas (AAMR: 12.1 vs. 10.1), particularly among females (AAMR: 5.9 vs. 3.8), while males had same AAMRs. These findings, consistent with Indian [8] and Nepal [31] studies, emphasize increasing rural healthcare funding for women, establishing mobile screening units and adolescent-friendly clinics, and leveraging local influencers to promote cancer screening and health-seeking behaviour among young women.

In Varanasi, the AAIR for all cancers among AYA is higher in males (22.2 per 100,000 population) than in females (19.5 per 100,000 population), largely due to the high incidence of oral cancer linked to tobacco and areca nut use among males. Conversely, across 28 Indian PBCRs, the median AAIR is higher in females (29.2 per 100,000 population) than in males (22.2 per 100,000 population), with breast, thyroid, gall bladder and cervical cancers contributing significantly to female incidence rates [7]. Similarly, cancer registries in the US [30] and Europe [20] report higher AAIRs in AYA females than males due to the prevalence of these cancers in females.

### Disparities in sociodemographic characteristics and cancer care between AYA patients and older adults

The multivariable analysis revealed significant socio-demographic and cancer-related disparities between AYA patients and those aged ≥ 40 years. AYAs had 2 to 5 times higher odds of better education but lower odds of employment and income compared to older patients. They were also more likely to have cancer confirmed microscopically, reflecting better diagnostic access linked to higher education and health literacy, which may enable timely care. AYAs were more likely to complete or be undergoing treatment, supported by their education and strong social networks, which can help overcome socio-economic challenges. However, higher wealth and job stability among older patients do not always translate to better healthcare utilization, as factors like geography, family background, and systemic inequalities also play key roles [32]. The lower income and employment status among AYAs, a critical working-age group, highlight their financial vulnerability during cancer treatment. Expanding financial assistance, patient navigation, peer support, and empowering AYAs with healthcare resources is essential to address these barriers and improve outcomes.

The overall study findings call for multilevel interventions: at the individual level, enhance awareness of risk factors and screening, especially for oral and breast cancers; at the community level, leverage peer networks, community leaders, and culturally sensitive education to promote behavior change like tobacco cessation; at the health system level, integrate AYA-specific cancer screening into primary care, train providers in early detection and culturally competent care, and implement patient navigation; and at the policy level, strengthen regulations such as COTPA, provide financial assistance, address environmental risks, and expand PBCR capacity for robust data.

### Limitations

This study, based on a newly established PBCR, faced limitations. We had limited follow-up information to analyse 5-year survival rates. Additionally, case ascertainment completeness indices indicated an under-registration of 10–20% in different district blocks, notably among rural, elderly, and pediatric populations, especially girls, due to disparities in service accessibility. Since we utilized only incidence-based data, we could not evaluate crucial variables such as cancer staging, health insurance, comorbidities, awareness information, and risk factors, including lifestyle and environmental factors. Consequently, these findings cannot be generalized to reflect nationwide disparities. Our analysis compared AYAs with individuals aged 40 years and above to highlight differences in sociodemographic characteristics and cancer care disparities. We recognize that grouping all individuals aged 40 and above into a single category encompasses a diverse population with varying life experiences and potentially distinct impacts from cancer. However, given the limited sample size and our focus on delineating the specific challenges of AYAs, further stratification of the older group was not feasible. This limitation should be considered when interpreting our findings. Future research with larger populations may allow for more granular analysis of older age subgroups.

## Conclusion

This study highlights the critical need for focused adolescent and young adult oncology in Indian settings, given the significant cancer burden among this age group in Varanasi, Uttar Pradesh. The data reveal distinct sex-specific, geographical disparities and social disparities in cancer incidence, mortality and care seeking. Effective cancer prevention and control policies must address these disparities through targeted health education, robust screening programs, and enhanced access to healthcare. Integrating cancer screening with adolescent health programs and enhancing support networks can improve early detection and treatment outcomes for AYA patients. Addressing socio-economic barriers and promoting cancer awareness is crucial for improving care utilization across demographic groups. The high burden of oral cancer among adolescents and young adults underscores urgent comprehensive action for tobacco control. This study underscores the importance of region-specific research in informing and formulating effective AYA-specific cancer prevention and control strategies.

## Supplementary Information

Below is the link to the electronic supplementary material.

Supplementary file1 (DOCX 90 KB)

## Data Availability

The datasets generated and/or analysed to support this study’s findings are contained within the Varanasi population-based cancer registry but are not publicly available due to confidentiality, security, and ownership matters. They may be available from the corresponding author upon reasonable request.
