# Supplementary material for: Epidemiology of adolescent and young adult cancer and associated disparities in cancer pattern and care in India: findings from Varanasi’s population-based cancer registry, 2017–2019
Source: Cancer Causes Control. 2025 Jul 12;36(11):1477–88. doi: 10.1007/s10552-025-02030-2 (PMC12578687; doi:10.1007/s10552-025-02030-2)
Supplement: Supplementary file 1 — Supplementary file1 (DOCX 90 KB) [file 10552_2025_2030_MOESM1_ESM.docx]

**SUPPLEMENTARY INFORMATION**

**Supplementary Table 1 Socio-demographic and Cancer Profile of Patients Aged 15-39 years (N=1105).**

| **Variables** | **Frequency** | **Percentage** |
| --- | --- | --- |
| Male | 604 | 54.7 |
| Female | 501 | 45.3 |
| **Educational Qualification** |  |  |
| Illiterate | 110 | 9.9 |
| Literate | 217 | 19.6 |
| Upto secondary | 507 | 45.8 |
| Senior secondary or higher | 254 | 22.9 |
| No information/unknown | 17 | 1.5 |
| **Residence** |  |  |
| Rural | 560 | 50.7 |
| Urban | 545 | 49.3 |
| **Religion** |  |  |
| Hindu | 923 | 83.5 |
| Others | 182 | 16.5 |
| **Mother Tongue** |  |  |
| Hindi | 1046 | 94.6 |
| Others | 59 | 5.4 |
| **Occupation** |  |  |
| Professional, semi-professional, clerical, government employee, private employee | 156 | 14.1 |
| Farmer, skilled worker, semi-skilled worker, unskilled worker, others | 398 | 36.0 |
| Unemployed, student, house-wife | 542 | 49.0 |
| No information | 9 | 0.8 |
| **Socio-economic Status** |  |  |
| Upper and upper middle | 130 | 11.7 |
| Lower middle | 353 | 31.9 |
| Lower | 501 | 45.3 |
| No information/unknown | 121 | 10.9 |
| **Primary Site Large Organs System** |  |  |
| Mouth | 182 | 16.5 |
| Breast | 135 | 12.2 |
| Tongue | 69 | 6.2 |
| Other Unspecified | 61 | 5.5 |
| Myeloid Leukaemia | 58 | 5.2 |
| Gallbladder | 49 | 4.4 |
| Cervix uteri | 46 | 4.2 |
| Rectum | 40 | 3.6 |
| Ovary | 40 | 3.6 |
| Bone | 39 | 3.5 |
| Liver | 37 | 3.3 |
| Brain, Nervous System | 35 | 3.2 |
| Colon | 28 | 2.5 |
| Leukaemia Unspecified | 25 | 2.3 |
| Trachea, Bronchus and Lung | 23 | 2.1 |
| Thyroid | 22 | 2.0 |
| Connective and Soft Tissue | 19 | 1.7 |
| Non-Hodgkin Lymphoma | 19 | 1.7 |
| Stomach | 18 | 1.6 |
| Hodgkin Disease | 17 | 1.5 |
| Lip | 15 | 1.4 |
| Testis | 13 | 1.2 |
| Lymphoid Leukemia | 12 | 1.1 |
| Kidney | 10 | 0.9 |
| Salivary Glands | 9 | 0.8 |
| Anus | 9 | 0.8 |
| Pancreas | 9 | 0.8 |
| Blader | 9 | 0.8 |
| Nose,sinuses | 7 | 0.6 |
| Larynx | 6 | 0.5 |
| Oesophagus | 5 | 0.5 |
| Nasopharynx | 3 | 0.3 |
| Other Thoracic organ | 3 | 0.3 |
| Corpus Uteri | 2 | 0.2 |
| Adrenal Gland | 2 | 0.2 |
| Multiple Myleoma | 2 | 0.2 |
| Tonsil | 1 | 0.1 |
| Other Oropharynx | 1 | 0.1 |
| Pharynx Unspecified | 1 | 0.1 |
| Small intestine | 1 | 0.1 |
| Melanoma of Skin | 1 | 0.1 |
| Vulva | 1 | 0.1 |
| Uterus Unspecified | 1 | 0.1 |
| Placenta | 1 | 0.1 |
| Penis | 1 | 0.1 |
| Prostate | 1 | 0.1 |
| Eye | 1 | 0.1 |
| No treatment | 33 | 3.0 |
| No information/unknown | 89 | 8.1 |
| **Basis of Diagnosis** |  |  |
| Death Certificate only | 5 | 0.5 |
| Clinical | 82 | 7.4 |
| Radiology | 88 | 8.0 |
| Cytology | 143 | 12.9 |
| Histology of Primary | 654 | 59.2 |
| Verbal Autopsy | 133 | 12.0 |
| **Treatment** |  |  |
| Surgery | 115 | 10.4 |
| Radiotherapy | 20 | 1.8 |
| Chemotherapy | 165 | 14.9 |
| Multi-modality | 469 | 42.4 |
| Other alternative system | 27 | 2.4 |
| Palliative | 187 | 16.9 |
| No Treatment | 33 | 3.0 |
| No Information/Unknown | 89 | 8.1 |
| **Treatment Status** |  |  |
| Complete | 230 | 20.8 |
| Ongoing | 190 | 17.2 |
| Not completed | 426 | 38.6 |
| Not applicable | 37 | 3.3 |
| No information/unknown | 222 | 20.1 |

**Supplementary Figure 1 Age-adjusted rates for cancer incidence and mortality (per 100 000 population) among the Adolescent and Young Adult cancer patients in Varanasi, 2017-2019**

**Supplementary Figure 2 Sex-wise age-adjusted rates for cancer incidence per 100 000 population among the different age groups in Adolescent and Young Adult cancer patients in Varanasi, 2017-2019**

**Supplementary Table 2 Average Annual Age Specific, Crude (CR), Age-adjusted (AAR) and Truncated Rates (15-39 years; TR) Incidence Rate per 100,000 Population: 2017-2019, both sexes, Varanasi District, 2017-2019**

| **ICD-O** | **Primary Site, overall** | **15-19** | **20-24** | **25-29** | **30-34** | **35-39** | **Total** | **CR** | **AAR** | **TR** |
| --- | --- | --- | --- | --- | --- | --- | --- | --- | --- | --- |
| **C00** | Lip | 0 | 0 | 3 | 6 | 6 | 15 | 0.3 | 0.1 | 0.3 |
| **C01-C02** | Tongue | 0 | 2 | 5 | 20 | 42 | 69 | 1.3 | 0.5 | 1.3 |
| **C03-C06** | Mouth | 3 | 8 | 22 | 51 | 98 | 182 | 3.3 | 1.3 | 3.5 |
| **C07-C08** | Salivary Glands | 0 | 2 | 2 | 3 | 2 | 9 | 0.2 | 0.1 | 0.2 |
| **C09** | Tonsil | 0 | 0 | 0 | 1 | 0 | 1 | 0.0 | 0.0 | 0.0 |
| **C10** | Other Oropharynx | 0 | 0 | 0 | 0 | 1 | 1 | 0.0 | 0.0 | 0.0 |
| **C11** | Nasopharynx | 1 | 1 | 1 | 0 | 0 | 3 | 0.1 | 0.0 | 0.1 |
| **C14** | Pharynx Unspecified | 0 | 0 | 0 | 0 | 1 | 1 | 0.0 | 0.0 | 0.0 |
| **C15** | Oesophagus | 0 | 0 | 0 | 2 | 3 | 5 | 0.1 | 0.0 | 0.1 |
| **C16** | Stomach | 0 | 3 | 4 | 7 | 4 | 18 | 0.3 | 0.1 | 0.3 |
| **C17** | Small intestine | 0 | 0 | 0 | 0 | 1 | 1 | 0.0 | 0.0 | 0.0 |
| **C18** | Colon | 3 | 7 | 5 | 2 | 11 | 28 | 0.5 | 0.2 | 0.5 |
| **C19** | Rectum | 5 | 4 | 10 | 10 | 11 | 40 | 0.7 | 0.3 | 0.8 |
| **C21** | Anus | 1 | 1 | 0 | 1 | 6 | 9 | 0.2 | 0.1 | 0.2 |
| **C22** | Liver | 1 | 8 | 8 | 3 | 17 | 37 | 0.7 | 0.3 | 0.7 |
| **C23-C24** | Gallbladder | 0 | 4 | 4 | 10 | 31 | 49 | 0.9 | 0.3 | 0.9 |
| **C25** | Pancreas | 0 | 1 | 1 | 2 | 5 | 9 | 0.2 | 0.1 | 0.2 |
| **C30-C31** | Nose, Sinuses | 2 | 1 | 1 | 1 | 2 | 7 | 0.1 | 0.0 | 0.1 |
| **C32** | Larynx | 1 | 0 | 0 | 1 | 4 | 6 | 0.1 | 0.0 | 0.1 |
| **C33-C34** | Trachea, Bronchus, and Lung | 1 | 3 | 2 | 6 | 11 | 23 | 0.4 | 0.2 | 0.4 |
| **C37-C38** | Other thoracic organ | 1 | 2 | 0 | 0 | 0 | 3 | 0.1 | 0.0 | 0.1 |
| **C40-C41** | Bone | 9 | 11 | 7 | 7 | 5 | 39 | 0.7 | 0.3 | 0.7 |
| **C43** | Melanoma of Skin | 0 | 0 | 0 | 1 | 0 | 1 | 0.0 | 0.0 | 0.0 |
| **C44,C47,C49** | Connective and Soft Tissue | 8 | 4 | 4 | 5 | 14 | 35 | 0.3 | 0.2 | 0.6 |
| **C50** | Breast | 1 | 6 | 22 | 47 | 59 | 135 | 2.5 | 1.0 | 2.6 |
| **C51** | Vulva | 0 | 0 | 0 | 1 | 0 | 1 | 0.0 | 0.0 | 0.0 |
| **C53** | Cervix Uteri | 0 | 6 | 8 | 10 | 22 | 46 | 0.8 | 0.3 | 0.9 |
| **C54** | Corpus Uteri | 0 | 0 | 0 | 0 | 2 | 2 | 0.0 | 0.0 | 0.0 |
| **C55** | Uterus Unspecified | 0 | 0 | 0 | 0 | 1 | 1 | 0.0 | 0.0 | 0.0 |
| **C56** | Ovary | 5 | 5 | 3 | 13 | 14 | 40 | 0.7 | 0.3 | 0.7 |
| **C58** | Placenta | 0 | 0 | 1 | 0 | 0 | 1 | 0.0 | 0.0 | 0.0 |
| **C60** | Penis | 0 | 0 | 0 | 0 | 1 | 1 | 0.0 | 0.0 | 0.0 |
| **C61** | Prostate | 0 | 0 | 0 | 0 | 1 | 1 | 0.0 | 0.0 | 0.0 |
| **C62** | Testis | 0 | 3 | 3 | 4 | 3 | 13 | 0.2 | 0.1 | 0.3 |
| **C64** | Kidney | 0 | 3 | 4 | 2 | 1 | 10 | 0.2 | 0.1 | 0.2 |
| **C67** | Blader | 0 | 1 | 1 | 2 | 5 | 9 | 0.2 | 0.1 | 0.2 |
| **C69** | Eye | 0 | 0 | 0 | 1 | 0 | 1 | 0.0 | 0.0 | 0.0 |
| **C70-C72** | Brain, Nervous system | 5 | 9 | 8 | 7 | 6 | 35 | 0.6 | 0.2 | 0.7 |
| **C73** | Thyroid | 1 | 5 | 5 | 2 | 9 | 22 | 0.4 | 0.2 | 0.4 |
| **C74** | Adrenal gland | 1 | 0 | 0 | 1 | 0 | 2 | 0.0 | 0.0 | 0.0 |
| **C81** | Hodgkin disease | 4 | 4 | 5 | 3 | 1 | 17 | 0.3 | 0.1 | 0.3 |
| **C82-C85, C96** | Non-Hodgkin lymphoma | 5 | 8 | 1 | 3 | 2 | 19 | 0.3 | 0.1 | 0.3 |
| **C90** | Multiple myeloma | 0 | 0 | 0 | 0 | 2 | 2 | 0.0 | 0.0 | 0.0 |
| **C91** | Lymphoid leukaemia | 1 | 4 | 2 | 4 | 1 | 12 | 0.2 | 0.1 | 0.2 |
| **C92-C94** | Myeloid leukaemia | 7 | 5 | 15 | 14 | 17 | 58 | 1.1 | 0.4 | 1.1 |
| **C95** | Leukaemia unspecified | 6 | 7 | 3 | 5 | 4 | 25 | 0.5 | 0.2 | 0.5 |
| **O&U*** | Other unspecified | 14 | 12 | 9 | 9 | 17 | 61 | 1.1 | 0.4 | 1.1 |
| **Total** |  | **8** | **140** | **169** | **267** | **443** | **1105** | **20.2** | **7.76** | **21.0** |

**Supplementary Table 3 Average Annual Age Specific, Crude (CR), Age-adjusted (AAR) and Truncated Rates (15-39 years; TR) Incidence Rate per 100,000 population: 2017-2019, Males, Varanasi District, 2017-2019**

| **ICD-O** | **Primary Site, Male** | **15-19** | **20-24** | **25-29** | **30-34** | **35-39** | **Total** | **CR** | **AAR** | **TR** |
| --- | --- | --- | --- | --- | --- | --- | --- | --- | --- | --- |
| **C00** | Lip | 0 | 0 | 3 | 6 | 6 | 15 | 0.5 | 0.2 | 0.6 |
| **C01-C02** | Tongue | 0 | 1 | 5 | 18 | 37 | 61 | 2.1 | 0.9 | 2.3 |
| **C03-C06** | Mouth | 3 | 8 | 22 | 44 | 88 | 165 | 5.8 | 2.3 | 6.3 |
| **C07-C08** | Salivary Glands | 0 | 2 | 1 | 2 | 1 | 6 | 0.2 | 0.1 | 0.2 |
| **C09** | Tonsil | 0 | 0 | 0 | 1 | 0 | 1 | 0.0 | 0.0 | 0.0 |
| **C11** | Nasopharynx | 0 | 1 | 0 | 0 | 0 | 1 | 0.0 | 0.0 | 0.0 |
| **C15** | Oesophagus | 0 | 0 | 0 | 1 | 1 | 2 | 0.1 | 0.0 | 0.1 |
| **C16** | Stomach | 0 | 1 | 2 | 2 | 0 | 5 | 0.2 | 0.1 | 0.2 |
| **C17** | Small Intestine | 0 | 0 | 0 | 0 | 1 | 1 | 0.0 | 0.0 | 0.0 |
| **C18** | Colon | 2 | 5 | 2 | 2 | 9 | 20 | 0.7 | 0.3 | 0.7 |
| **C19** | Rectum | 5 | 2 | 7 | 7 | 6 | 27 | 0.9 | 0.4 | 1.0 |
| **C21** | Anus | 1 | 0 | 0 | 1 | 2 | 4 | 0.1 | 0.1 | 0.1 |
| **C22** | Liver | 0 | 2 | 5 | 2 | 7 | 16 | 0.6 | 0.2 | 0.6 |
| **C23-C24** | Gallbladder | 0 | 2 | 2 | 3 | 5 | 12 | 0.4 | 0.2 | 0.5 |
| **C25** | Pancreas | 0 | 1 | 1 | 1 | 4 | 7 | 0.2 | 0.1 | 0.3 |
| **C30-C31** | Nose, Sinuses etc | 2 | 1 | 1 | 0 | 2 | 6 | 0.2 | 0.1 | 0.2 |
| **C32** | Larynx | 0 | 0 | 0 | 1 | 2 | 3 | 0.1 | 0.0 | 0.1 |
| **C33-C34** | Trachea, Bronchus and Lung | 1 | 3 | 0 | 4 | 7 | 15 | 0.5 | 0.2 | 0.5 |
| **C37-C38** | Other Thoracic Organ | 1 | 2 | 0 | 0 | 0 | 3 | 0.1 | 0.0 | 0.1 |
| **C40-C41** | Bone | 7 | 10 | 4 | 3 | 5 | 29 | 1.0 | 0.4 | 1.0 |
| **C44,C47,C49** | Connective and Soft Tissue | 3 | 3 | 1 | 3 | 6 | 16 | 0.2 | 0.2 | 0.6 |
| **C60** | Penis | 0 | 0 | 0 | 0 | 1 | 1 | 0.0 | 0.0 | 0.0 |
| **C61** | Prostate | 0 | 0 | 0 | 0 | 1 | 1 | 0.0 | 0.0 | 0.0 |
| **C62** | Testis | 0 | 3 | 3 | 4 | 3 | 13 | 0.5 | 0.2 | 0.5 |
| **C64** | Kidney | 0 | 2 | 1 | 2 | 0 | 5 | 0.2 | 0.1 | 0.2 |
| **C67** | Blader | 0 | 1 | 0 | 2 | 2 | 5 | 0.2 | 0.1 | 0.2 |
| **C69** | Eye | 0 | 0 | 0 | 1 | 0 | 1 | 0.0 | 0.0 | 0.0 |
| **C70-C72** | Brain, Nervous system | 3 | 7 | 5 | 6 | 4 | 25 | 0.9 | 0.3 | 0.9 |
| **C73** | Thyroid | 0 | 3 | 1 | 0 | 1 | 5 | 0.2 | 0.1 | 0.2 |
| **C81** | Hodgkin Disease | 3 | 3 | 2 | 3 | 1 | 12 | 0.4 | 0.2 | 0.4 |
| **C82-C85,C96** | Non-Hodgkin Lymphoma | 3 | 8 | 1 | 2 | 0 | 14 | 0.5 | 0.2 | 0.5 |
| **C90** | Multiple Myeloma | 0 | 0 | 0 | 0 | 2 | 2 | 0.1 | 0.0 | 0.1 |
| **C91** | Lymphoid Leukaemia | 1 | 4 | 2 | 4 | 1 | 12 | 0.4 | 0.2 | 0.4 |
| **C92-C94** | Myeloid Leukaemia | 5 | 3 | 10 | 9 | 7 | 34 | 1.2 | 0.5 | 1.3 |
| **C95** | Leukaemia Unspecified | 5 | 6 | 1 | 1 | 4 | 17 | 0.6 | 0.2 | 0.6 |
| **O&U** | Other Unspecified | 9 | 9 | 5 | 4 | 15 | 42 | 1.5 | 0.6 | 1.5 |
| **Total** | | **54** | **93** | **87** | **139** | **231** | **604** | **21.1** | **8.2** | **22.2** |

**Supplementary Table 4 Average Annual Age Specific, Crude (CR), Age-adjusted (AAR) and Truncated Rates (15-39 years; TR) Incidence Rate per 100,000 Population: 2017-2019, Females, Varanasi district, 2017-2019**

| **ICD-O** | **Primary Site, Female** | **15-19** | **20-24** | **25-29** | **30-34** | **35-39** | **Total** | **CR** | **AAR** | **TR** |
| --- | --- | --- | --- | --- | --- | --- | --- | --- | --- | --- |
| **C01-C02** | Tongue | 0 | 1 | 0 | 2 | 5 | 8 | 0.3 | 0.1 | 0.3 |
| **C03-C06** | Mouth | 0 | 0 | 0 | 7 | 10 | 17 | 0.6 | 0.2 | 0.6 |
| **C07-C08** | Salivary Glands | 0 | 0 | 1 | 1 | 1 | 3 | 0.1 | 0.0 | 0.1 |
| **C10** | Other Oropharynx | 0 | 0 | 0 | 0 | 1 | 1 | 0.0 | 0.0 | 0.0 |
| **C11** | Nasopharynx | 1 | 0 | 1 | 0 | 0 | 2 | 0.1 | 0.0 | 0.1 |
| **C14** | Pharynx Unspecified | 0 | 0 | 0 | 0 | 1 | 1 | 0.0 | 0.0 | 0.0 |
| **C15** | Oesophagus | 0 | 0 | 0 | 1 | 2 | 3 | 0.1 | 0.0 | 0.1 |
| **C16** | Stomach | 0 | 2 | 2 | 5 | 4 | 13 | 0.5 | 0.2 | 0.5 |
| **C18** | Colon | 1 | 2 | 3 | 0 | 2 | 8 | 0.3 | 0.1 | 0.3 |
| **C19** | Rectum | 0 | 2 | 3 | 3 | 5 | 13 | 0.5 | 0.2 | 0.5 |
| **C21** | Anus | 0 | 1 | 0 | 0 | 4 | 5 | 0.2 | 0.1 | 0.2 |
| **C22** | Liver | 1 | 6 | 3 | 1 | 10 | 21 | 0.8 | 0.3 | 0.8 |
| **C23-C24** | Gallbladder | 0 | 2 | 2 | 7 | 26 | 37 | 1.4 | 0.5 | 1.4 |
| **C25** | Pancreas | 0 | 0 | 0 | 1 | 1 | 2 | 0.1 | 0.0 | 0.1 |
| **C30-C31** | Nose, Sinuses etc | 0 | 0 | 0 | 1 | 0 | 1 | 0.0 | 0.0 | 0.0 |
| **C32** | Larynx | 1 | 0 | 0 | 0 | 2 | 3 | 0.1 | 0.0 | 0.1 |
| **C33-C34** | Trachea, Bronchus and Lung | 0 | 0 | 2 | 2 | 4 | 8 | 0.3 | 0.1 | 0.3 |
| **C40-C41** | Bone | 2 | 1 | 3 | 4 | 0 | 10 | 0.4 | 0.1 | 0.4 |
| **C43** | Melanoma of Skin | 0 | 0 | 0 | 1 | 0 | 1 | 0.0 | 0.0 | 0.0 |
| **C47,C49** | Connective and Soft tissue | 5 | 1 | 3 | 2 | 8 | 19 | 0.5 | 0.3 | 0.7 |
| **C50** | Breast | 1 | 6 | 22 | 47 | 59 | 135 | 5.2 | 2.0 | 5.3 |
| **C51** | Vulva | 0 | 0 | 0 | 1 | 0 | 1 | 0.0 | 0.0 | 0.0 |
| **C53** | Cervix Uteri | 0 | 6 | 8 | 10 | 22 | 46 | 1.8 | 0.7 | 1.8 |
| **C54** | Corpus Uteri | 0 | 0 | 0 | 0 | 2 | 2 | 0.1 | 0.0 | 0.1 |
| **C55** | Uterus Unspecified | 0 | 0 | 0 | 0 | 1 | 1 | 0.0 | 0.0 | 0.0 |
| **C56** | Ovary | 5 | 5 | 3 | 13 | 14 | 40 | 1.5 | 0.6 | 1.5 |
| **C58** | Placenta | 0 | 0 | 1 | 0 | 0 | 1 | 0.0 | 0.0 | 0.0 |
| **C64** | Kidney | 0 | 1 | 3 | 0 | 1 | 5 | 0.2 | 0.1 | 0.2 |
| **C67** | Blader | 0 | 0 | 1 | 0 | 3 | 4 | 0.2 | 0.1 | 0.2 |
| **C70-C72** | Brain, Nervous System | 2 | 2 | 3 | 1 | 2 | 10 | 0.4 | 0.1 | 0.4 |
| **C73** | Thyroid | 1 | 2 | 4 | 2 | 8 | 17 | 0.6 | 0.2 | 0.7 |
| **C74** | Adrenal gland | 1 | 0 | 0 | 1 | 0 | 2 | 0.1 | 0.0 | 0.1 |
| **C81** | Hodgkin Disease | 1 | 1 | 3 | 0 | 0 | 5 | 0.2 | 0.1 | 0.2 |
| **C82-C85,C96** | Non-Hodgkin Lymphoma | 2 | 0 | 0 | 1 | 2 | 5 | 0.2 | 0.1 | 0.2 |
| **C92-C94** | Myeloid Leukaemia | 2 | 2 | 5 | 5 | 10 | 24 | 0.9 | 0.3 | 0.9 |
| **C95** | Leukaemia Unspecified | 1 | 1 | 2 | 4 | 0 | 8 | 0.3 | 0.1 | 0.3 |
| **O&U** | Other Unspecified | 5 | 3 | 4 | 5 | 2 | 19 | 0.7 | 0.3 | 0.7 |
| **Total** |  | **32** | **47** | **82** | **128** | **212** | **501** | **19.1** | **7.2** | **19.5** |

**Supplementary Table 5 Incidence rate (AAR per 100 000) and Cumulative risk (% and 1 in person) of Leading Cancer Sites Among Male Patients Aged 15-39 years, Varanasi, India, 2017-2019. (N=604)**

| **Primary Site** | **AAR** | **Truncated rates** | **Cum Risk**  **(15-39) %** | **1 in Persons** |
| --- | --- | --- | --- | --- |
| Mouth | 2.3 | 6.3 | 0.00212 | 472 |
| Tongue | 0.9 | 2.3 | 0.00080 | 1244 |
| Other Unspecified | 0.6 | 1.5 | 0.00047 | 2144 |
| Myeloid Leukaemia | 0.5 | 1.3 | 0.00039 | 2532 |
| Rectum | 0.4 | 1.0 | 0.00031 | 3210 |
| Bone | 0.4 | 1.0 | 0.00030 | 3331 |
| Brain, Nervous System | 0.3 | 0.9 | 0.00028 | 3589 |
| Colon | 0.3 | 0.7 | 0.00023 | 4301 |
| Liver | 0.2 | 0.6 | 0.00020 | 5092 |
| Lip | 0.2 | 0.6 | 0.00019 | 5138 |
| Trachea, Bronchus and Lung | 0.2 | 0.5 | 0.00018 | 5468 |
| Leukaemia Unspecified | 0.2 | 0.6 | 0.00017 | 5768 |
| Connective and Soft Tissue | 0.2 | 0.6 | 0.00017 | 5965 |
| Testis | 0.2 | 0.5 | 0.00016 | 6440 |
| Gallbladder | 0.2 | 0.5 | 0.00015 | 6739 |
| Non-Hodgkin Lymphoma | 0.2 | 0.5 | 0.00014 | 7394 |
| Lymphoid Leukaemia | 0.2 | 0.4 | 0.00013 | 7463 |
| Hodgkin Disease | 0.2 | 0.4 | 0.00013 | 7850 |
| Pancreas | 0.1 | 0.3 | 0.00009 | 11389 |
| Salivary glands | 0.1 | 0.2 | 0.00007 | 14329 |
| Nose, sinuses | 0.1 | 0.2 | 0.00006 | 15769 |
| Bladder | 0.1 | 0.2 | 0.00006 | 15962 |
| Stomach | 0.1 | 0.2 | 0.00006 | 17088 |
| Kidney | 0.1 | 0.2 | 0.00006 | 17761 |
| Thyroid | 0.1 | 0.2 | 0.00005 | 19044 |
| Anus | 0.1 | 0.1 | 0.00005 | 20781 |
| Larynx | 0.0 | 0.1 | 0.00004 | 24870 |
| Multiple Myeloma | 0.0 | 0.1 | 0.00003 | 37078 |
| Oesophagus | 0.0 | 0.1 | 0.00003 | 37419 |
| Other Thoracic Organ | 0.0 | 0.1 | 0.00003 | 38003 |
| Small Intestine | 0.0 | 0.0 | 0.00001 | 74156 |
| Penis | 0.0 | 0.0 | 0.00001 | 74156 |
| Prostate | 0.0 | 0.0 | 0.00001 | 74156 |
| Tonsil | 0.0 | 0.0 | 0.00001 | 75533 |
| Eye | 0.0 | 0.0 | 0.00001 | 75533 |
| Nasopharynx | 0.0 | 0.0 | 0.00001 | 108684 |
| **Total** | **8.2** | **22.2** | **0.00723** | **138** |

**Supplementary Table 6 Incidence rate (AAR per 100 000) and Cumulative risk (% and 1 in person) of Leading Cancer Sites Among Female Patients Aged 15-39 years, Varanasi, India, 2017-2019. (N=501)**

| **Primary Site** | **AAR** | **Truncated rates** | **Cum Risk**  **(15-39) %** | **1 in Persons** |
| --- | --- | --- | --- | --- |
| Breast | 2.0 | 5.3 | 0.00179 | 559 |
| Cervix Uteri | 0.7 | 1.8 | 0.00060 | 1659 |
| Ovary | 0.6 | 1.5 | 0.00050 | 1986 |
| Gallbladder | 0.5 | 1.4 | 0.00050 | 1998 |
| Myeloid Leukaemia | 0.3 | 0.9 | 0.00031 | 3267 |
| Liver | 0.3 | 0.8 | 0.00026 | 3792 |
| Mouth | 0.2 | 0.6 | 0.00023 | 4305 |
| Other Unspecified | 0.3 | 0.7 | 0.00022 | 4540 |
| Thyroid | 0.2 | 0.7 | 0.00022 | 4602 |
| Connective and Soft Tissue | 0.3 | 0.7 | 0.00022 | 4637 |
| Stomach | 0.2 | 0.5 | 0.00017 | 5923 |
| Rectum | 0.2 | 0.5 | 0.00017 | 5948 |
| Bone | 0.1 | 0.4 | 0.00012 | 8430 |
| Brain, Nervous System | 0.1 | 0.4 | 0.00012 | 8526 |
| Tongue | 0.1 | 0.3 | 0.00011 | 9335 |
| Trachea, Bronchus and Lung | 0.1 | 0.3 | 0.00011 | 9382 |
| Leukaemia Unspecified | 0.1 | 0.3 | 0.00010 | 10236 |
| Colon | 0.1 | 0.3 | 0.00010 | 10508 |
| Anus | 0.1 | 0.2 | 0.00007 | 15035 |
| Kidney | 0.1 | 0.2 | 0.00006 | 16262 |
| Non-Hodgkin Lymphoma | 0.1 | 0.2 | 0.00006 | 17068 |
| Hodgkin Disease | 0.1 | 0.2 | 0.00006 | 17738 |
| Blader | 0.1 | 0.2 | 0.00005 | 18595 |
| Oesophagus | 0.0 | 0.1 | 0.00004 | 24330 |
| Salivary Glands | 0.0 | 0.1 | 0.00004 | 25362 |
| Larynx | 0.0 | 0.1 | 0.00004 | 27421 |
| Corpus Uteri | 0.0 | 0.1 | 0.00003 | 36069 |
| Pancreas | 0.0 | 0.1 | 0.00003 | 36712 |
| Adrenal Gland | 0.0 | 0.1 | 0.00002 | 45206 |
| Nasopharynx | 0.0 | 0.1 | 0.00002 | 47769 |
| Other Oropharynx | 0.0 | 0.0 | 0.00001 | 72139 |
| Pharynx Unspecified | 0.0 | 0.0 | 0.00001 | 72139 |
| Uterus Unspecified | 0.0 | 0.0 | 0.00001 | 72139 |
| Nose, Sinuses | 0.0 | 0.0 | 0.00001 | 74754 |
| Melanoma of Skin | 0.0 | 0.0 | 0.00001 | 74754 |
| Vulva | 0.0 | 0.0 | 0.00001 | 74754 |
| Placenta | 0.0 | 0.0 | 0.00001 | 82033 |
| **Total** | **7.2** | **19.5** | **0.00645** | **155** |

**Supplementary Figure 4 Age-Standardized Rates (ASIR; World) of oral cancer (C00-C06) among the Varanasi Adolescent and Young Adult males compared with that of the top-fifteen countries with the highest burden of oral cancer**
